# Inter-Varietal Variation in Phenolic Profile, Antioxidant, Anti-Inflammatory and Analgesic Activities of Two *Brassica rapa* Varieties: Influence on Pro-Inflammatory Mediators

**DOI:** 10.3390/molecules29010117

**Published:** 2023-12-24

**Authors:** Nida Nazar, Abdullah Ijaz Hussain, Hassaan Anwer Rathore

**Affiliations:** 1 Department of Chemistry, Government College University Faisalabad, Faisalabad 38000, Pakistan; nidanazar786@gmail.com; 2 Hi-Tech Lab, Government College University Faisalabad, Faisalabad 38000, Pakistan; 3 Department of Pharmaceutical Sciences, College of Pharmacy, QU Health, Qatar University, Doha 2713, Qatar

**Keywords:** polyphenols, carrageenan, C-reactive protein, TNF-α, IL-6, rheumatoid factor

## Abstract

The present research study aims to appraise the potential of polyphenol-rich extracts from two *Brassica rapa* varieties on antioxidant, anti-inflammatory and analgesic activities using carrageenan-induced paw edema model in rats. Methanol extracts of peels and pulps of *Brassica rapa* yellow root (BRYR) and *Brassica rapa* white root (BRWR) were prepared using the soxhlet extraction technique. All four extracts were analyzed by reversed-phase high-pressure liquid chromatography (RP-HPLC) for the polyphenols, and results showed that 10 phenolic acids and 4 flavonoids were detected. Gallic acid was the major phenolic acid (174.6–642.3 mg/100 g of dry plant material) while catechin was the major (34.45–358.5 mg/100 g of dry plant material) flavonoid detected in the extracts. The total phenolic contents (TPC) of BRYR peel, BRWR peel, BRYR pulp and BRWR pulp extracts were in the range of 1.21–5.01 mg/g of dry plant material, measured as GAE, whereas the total flavonoid contents (TFC) were found in the range of 0.90–3.95 mg/g of dry plant material, measured as QE. BRYR peel extract exhibited the best DPPH radical scavenging activity (IC_50_, 3.85 µg/mL) and reducing potential as compared with other extracts. The in vivo anti-inflammatory potential was assessed by carrageenan-induced rat paw edema, and the analgesic potential was investigated by a hot plate test. Suppression of biochemical inflammatory biomarkers including C-reactive protein (CRP), rheumatoid factor (RF) and tumor necrosis factor (TNF-α), and interleukin-6 (IL-6) concentration were also determined. Results showed that BRYR peel extracts reduced paw edema and suppressed the production of TNF-α, IL-6, CRP and RF most significantly, followed by BRWR peel, BRYR pulp and BRWR pulp extracts. In addition, histopathology observation also supports the anti-inflammatory effect of peel extracts as being greater than that of root pulp extracts. Moreover, it was observed that the analgesic effect of the root-peel extracts was also more pronounced as compared with root-pulp extracts. It can be concluded that BRYR peel extract has higher phenolic contents and showed higher suppression of TNF-α, IL-6, CRP and RF, with strong antioxidant, anti-inflammatory and analgesic effects.

## 1. Introduction

Inflammation is the main protective response of the primary immune system against damage and is characterized by both acute and chronic inflammations [1,2]. Acute inflammation results in the formation of edema, leukocyte infiltration, and macrophage infiltration in the damaged muscle [3]. Whereas, in chronic inflammation, prolonged infiltration of these immune system components results in more serious conditions such as arthritis, cancer, asthma, atherosclerosis, and autoimmune ailments [4]. The majority of health issues only appear when the inflammation persists for a long time and is irreversible. Inflammation triggers cytokine production, including immunosuppressive interleukin (IL-) 4 and IL-10, or the pro-carcinogenic IL-6 and TNF-α [5]. Furthermore, C-reactive protein (CRP) is a highly inflammatory protein that increases 1000 times at the site of injury or inflammation [6]. Therefore, CRP shows a vital impact on inflammatory processes and stimulates macrophages to secrete pro-inflammatory cytokines including tumor necrosis factor-α (TNF-α) and interleukin (IL) [7]. The eruption of pro-inflammatory cytokines (TNF-α and IL-6) from active inflammatory pathways tangled in pathological pain processes is essential for autoimmune disorders. Imbalance of pro-inflammatory cytokines and anti-inflammatory cytokines causes inflammation [5,6,7].

Non-steroidal anti-inflammatory drugs (NSAIDs) belong to an important class of pain-relieving drugs and suppress pro-inflammatory cytokines (CRP, TNF-α, and IL-6) with anti-inflammatory and analgesic effects [8]. However, existing NSAIDs and cyclooxygenase-2 (COX-2) inhibitors have also been linked with several adverse effects such as cardiovascular and abdominal complications [9]. Certain dietary polyphenols such as quercetin and catechin are natural antioxidants and exhibit the effect of balancing the generation of pro- and anti-inflammatory cytokines, and increase IL-10 secretion while inhibiting TNF-α [10,11]. Thus, anti-inflammatory drugs derived from natural sources and used in traditional medicine have attracted the attention of health professionals and the pharmaceutical industry due to their effectiveness and fewer side effects [1,2,4]. Polyphenols are a class of natural compounds that are under consideration due to many reasons [8,12,13].

*Brassica rapa* (*B. rapa*), commonly known as turnip, is an edible root consumed as a vegetable, which belongs to the Brassicaceae family [14,15,16]. *B. rapa* is a rich source of bioactive compounds including isothiocyanates, glucosinolates, anthocyanins, carotenoids, flavonoids, sulforaphane, organic acids and phenolic compounds [17,18]. The whole plant has been used not only for nourishment, but also as a traditional remedy against several diseases, including inflammation [2]. *B. rapa* ethanol extract has been reported to display an anti-arthritis effect against complete Freund’s adjuvant-induced inflammation and exhibits incredible anti-inflammatory effect and analgesic potential [2,19].

Inter-varietal comparative study of polyphenols-rich extracts of root-peel and root-pulp of yellow and white varieties of *Brassica rapa* with antioxidant, anti-inflammatory and analgesic effects on suppression of TNF-α, IL-6, CRP and RF are not reported yet. Therefore, the current study is designed to investigate the suppression of TNF-α, IL-6, CRP and RF levels in rats with antioxidant, anti-inflammatory and analgesic effects of polyphenol-rich extracts of root-peel and root-pulp of yellow and white varieties of *B. rapa*. Moreover, the extracts were analyzed for phenolic profile using reverse-phase high performance liquid chromatography (RP-HPLC).

## 2. Results and Discussion

### 2.1. Extract Yield

Table 1 shows the extract yields of BRYR peel, BRWR peel, BRYR pulp and BRWR pulp. The extracts’ yields ranged from 6.72 to 13.35 g/100 g of dry plant material. Peel extracts of *B. rapa* reported a lower yield compared with root pulp extracts. Overall, the yield of BRYR pulp extract was higher (13.35 g/100 g) than that of the other extracts. This study reported a significant difference (*p* ≤ 0.05) in *B. rapa* peel and root pulp extract yields, which may be due to differences in the composition of extractable constituents in different plant materials. Sultana et al. [20] reported a higher extract yield of yellow and white *Brassica rapa* than our findings, and the variation may be due to the variation in the extraction process. Extract yield depends on various factors, including the solvent polarity, method of extraction and time of the extraction procedure [12,20].

### 2.2. Quantification of Phenolic Acids and Flavonoid by RP-HPLC

**Figure 1:**
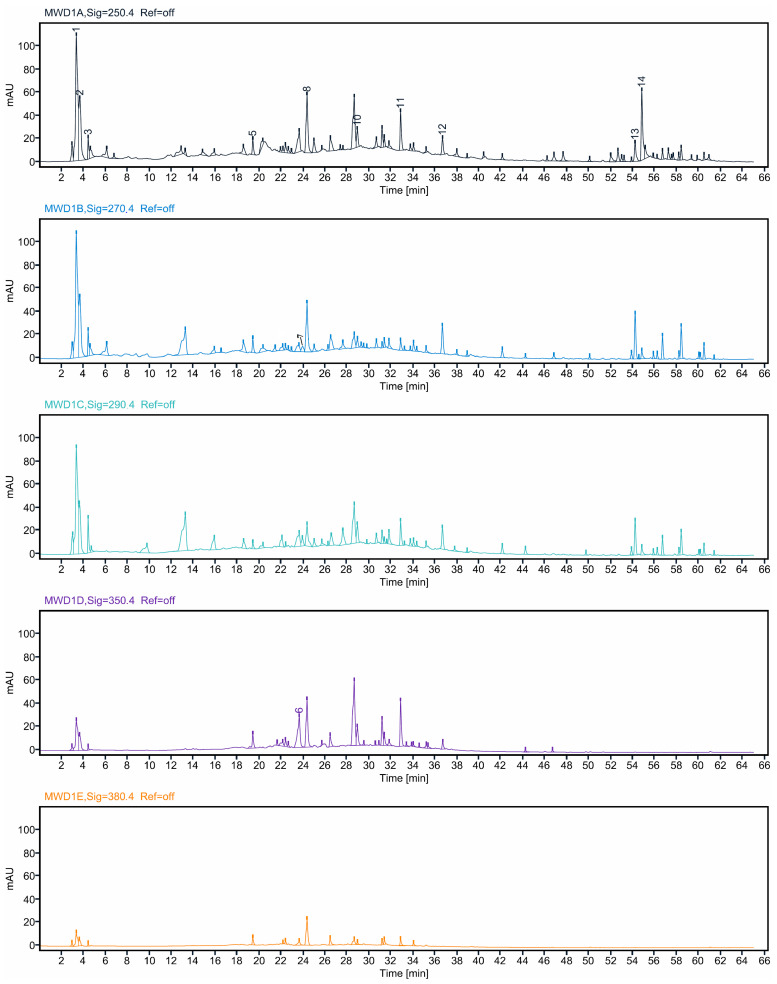
Chromatograms showing the separation of phenolic acids and flavonoids in *B. rapa* yellow peel extract at different wavelengths. Peak numbers represents the specific compounds as mentioned in Table 2.

The phenolic acid and flavonoid compositions of BRYR peel, BRWR peel, BRYR pulp, and BRWR pulp extracts were determined by RP-HPLC using a multi-wavelength detector (MWD). Chromatograms showing the separation of phenolic acids and flavonoids from the BRYR peel extract at 250, 270, 290, 350 and 380 nm are presented in Figure 1. The peak numbers mentioned are the compounds detected in the BRYR peel extract. Chromatograms of other extracts are presented in Supplementary Data. Qualitative and quantitative data from all the extracts are represented in Table 2. Ten phenolic acids were detected: gallic acid, chlorogenic acid, *p*-hydroxyl benzoic acid, syringic acid, vanillic acid, *p*-coumaric acid, sinapic acid, ferulic acid, cinnamic acid, and benzoic acid. Gallic acid (174.6–642.3 mg/100 g) and chlorogenic acid (84.3–267.1 mg/100 g) were the major phenolic acids present in abundance in all extracts. The BRYR peel extract showed the highest concentration of gallic acid, syringic acid, cinnamic acid and ferulic acid. Chlorogenic acid was in highest concentration in the pulp extracts of both BRWR and BRYR. Four flavonoids—catechin, kaempferol, quercetin and rutin—are being reported as the major polyphenols in *B. rapa* extracts. Catechin was reported to have the highest concentration in BRYR extract (358.5 mg/100 g) and the lowest (34.45 mg/100 g) in BRYR extract. Quercetin and kaempferol were reported in all the *B. rapa* extracts. The highest quercetin and kaempferol contents were reported in the BRWR peel extract, i.e., 11.38 and 34.21 mg/100 g, respectively. Rutin (4.63 mg/100 g) was only detected in BRYR pulp extract. The different *B. rapa* extracts retained altered phenolic acid and flavonoid concentrations, and a significant (*p* ≤ 0.05) difference was observed. Most polyphenol compounds have the ability to act as antioxidants and are polar in nature, and methanol and other polar solvents are the efficient solvents for the extraction of these phenolic acids and flavonoid compounds [21]. Therefore, the high concentrations of gallic acid, chlorogenic acid and *p*-coumeric acid in the analyzed extracts might be due to the polarity of the organic extraction solvent [12,13,20]. RP-HPLC investigation of BRYR peel, BRWR peel, BRYR pulp, and BRWR pulp extracts agrees well with findings stated by previous researchers [22,23,24,25,26,27,28]. The glycosylated derivatives of quercetin, kaempferol, and isorhamnetin have been reported in turnip top [22,24]. In another study, ferulic acid, sinapic acid, 3-p-coumaroylquinic and derivatives of kaempferol and isorhamnetin were identified in aqueous extracts of different parts of turnip [23]. Quercetin, kaempferol, hydroxybenzoic acid and hydroxycinnamic acid derivatives have also been reported in microgreens of *Brassica rapa* [27].

### 2.3. TPC, TFC and Antioxidant Activity

Total phenolic contents (TPC), total flavonoid contents (TFC), and DPPH radical scavenging activity of BRYR peel, BRWR peel, BRYR pulp and BRWR pulp extracts were determined and are presented in Table 1. Total phenolic contents were measured in the range of 1.21–5.01 mg/g of dry material as gallic acid equivalent (GAE) in all the *B. rapa* extracts. Generally, peel extracts showed the highest concentration of TPC as compared to pulp extract. Similarly, BRYR peel and BRWR peel extracts showed TPC 3.95 and 2.28 mg/g of dry material, measured as quercetin equivalents (QE). Overall, significantly (*p* ≤ 0.05) higher TPC (5.01 mg/g, GAE) and TFC (3.95 mg/g, QE) were observed in the BRYR peel extract, but these were lowest in the BRWR pulp extract (Table 1). The function of phenolic acids and flavonoid compounds include defense against free radicals, ROS, allergies, microorganisms, viruses, ulcers, tumors and inflammation [9]. The current results show that *Brassica rapa* extracts have higher TPC and TFC values as compared with those stated by Sultana et al. [20]. However, in another finding, aqueous extract of *Brassica rapa* root extract was reported to have TPC (9.41, GAE/mg) and TFC (1.01 µg QE/mg) values which were in partial agreement with our findings [29]. Yucetepe [30] also investigated the TPC (169.29 mg/g, GAE dry weight) in the purple peel of an extract of *Brassica rapa,* extracted with 80% methanol using ultrasonic extraction. These changes in TPC and TFC values can be due to different geographical, agricultural and climatic conditions of the regions.

A stable free radical, 2-2′-diphenyl-1-picrylhydrazyl (DPPH), was utilized to assess free radical scavenging ability of BRYR peel, BRWR peel, BRYR pulp and BRWR pulp extracts. Different concentrations of the extracts of *B. rapa* showed different radical scavenging activity, and the results are reported in terms of IC_50_ (µg/mL) in Table 1. IC_50_ values of the BRYR peel, BRWR peel, and BRYR pulp and BRWR pulp ranged between 3.85 and 5.76 µg/mL, as presented in Table 2. Antioxidant compounds found in plants, such as phenols and flavonoids, have been shown to protect against ROS and free radical damage [31]. A lower IC_50_ value of extract indicates a greater antioxidant activity of DPPH assay, and vice versa [21]. The BRYR peel extract exhibited the highest antioxidant potential (IC_50_, 3.85 µg/mL), while BRWR pulp showed the lowest antioxidant activity (IC_50_, 5.76 µg/mL). The synthetic antioxidant BHT showed the best DPPH radical scavenging activity (IC_50_ = 1.24 µg/mL). Therefore, the current study demonstrated that peel and pulp extracts of both yellow and white varieties extracts displayed potent antioxidant activity. The results of the present study on DPPH and reducing power tests were comparable to those of Saeed et al. [15]. Fernandes et al. [23] revealed that aqueous root extract of *Brassica rapa* was shown to have poor antioxidant capacity as compared to leaves, stems and flowers. However, these results are inconsistent with other studies showing that aqueous root extract of *B. rapa* has a better DPPH screening activity than the turnip green aqueous extract [32]. According to Berdja et al. [29], aqueous root extract of turnip was shown to have significantly lower DPPH antioxidant activity (2100 µg/mL) as compared to synthetic antioxidants. The difference in DPPH values compared to previous study results may be due to adopting altered extraction methods and differences in seasonal, agro-climatic and geographical conditions.

**Figure 2:**
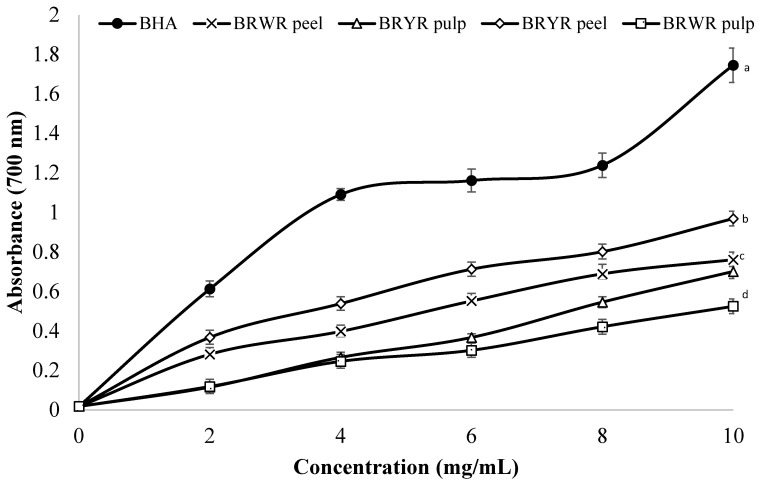
The reducing power assay of *B. rapa* extracts. Values are mean ± standard deviation of triplicate experiments. Superscripted different letters indicate the significant difference (*p* ≤ 0.05) between different extracts. BRYR peel: *B. rapa* yellow root peel; BRWR peel: *B. rapa* white root peel; BRYR pulp: *B. rapa* yellow root pulp; BRWR pulp: *B. rapa* white root pulp.

The reducing potential of BRYR peel, BRWR peel, BRYR pulp and BRWR pulp extracts were examined, and the results are presented in Figure 2. The different extracts of *B. rapa* (concentration up to 10.0 mg/mL) exhibited a concentration-dependent reducing power. Moreover, it is observed that different methanol extracts at 10 mg/mL showed some variation in reducing power that showed significant difference (*p* ≤ 0.05). A higher reducing power indicates the greater antioxidant potential of reducing power assay [21]. Among all the extracts, BRYR peel extract was better in terms of reducing power followed by BRWR peel, BRYR pulp and BRWR pulp extracts. The reducing power results of this study were comparable with those of previous studies investigated by other researchers [15,20,33]. Phytochemical characterization of peel extracts, especially yellow peel extracts, revealed the occurrence of more phenolic and flavonoid compounds. For reducing efficiency, the higher reduction power is due to the high phenolic contents [15].

### 2.4. Acute Inflammatory Model

**Figure 3:**
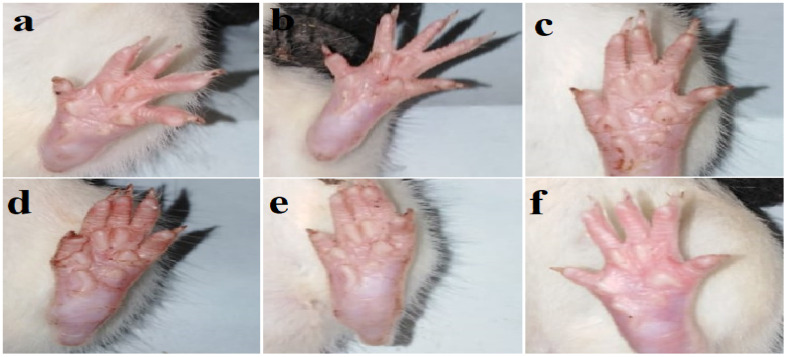
Representative images of rat’s paws after carrageenan injection. (**a**) Normal control group; (**b**) BRYR-Peel group (*B. rapa* yellow root peel); (**c**) BRWR-Peel group (*B. rapa* white root peel); (**d**) BRYR-Pulp group (*B. rapa* white root pulp); (**e**) BRWR-Pulp group (*B. rapa* white root pulp); (**f**) Positive control group (Indomethacin).

**Figure 4:**
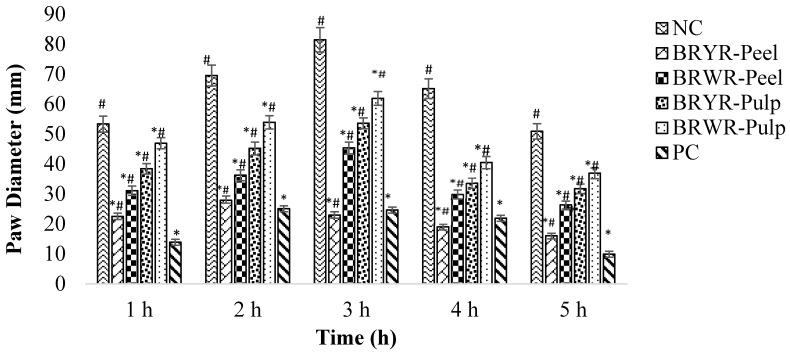
Effect of polyphenol-rich *B. rapa* extracts and indomethacin on paw diameter after carrageenan injection. NC group: Normal control; BRYR-Peel group: *B. rapa* yellow root peel; BRWR-Peel group: *B. rapa* white root peel; BRYR-Pulp group: *B. rapa* yellow root pulp; BRWR-Pulp group: *B. rapa* white root pulp; PC: Positive control group. * symbolizes significant difference (*p* ≤ 0.05) as compared to NC. # symbolizes significant difference (*p* ≤ 0.05) as compared to PC.

The effect of polyphenol-rich BRYR peel, BRWR peel, BRYR pulp and BRWR pulp extracts and indomethacin on carrageenan-induced paw edema is presented in Table 3. *B. rapa* extracts significantly reduced carrageenan-induced inflammation by decreasing the paw diameter of rats (Figure 3). In the present study, maximum inhibition of paw diameter was observed at 3 h after carrageenan injection (Figure 4). During the third observation hour (h), BRYR-peel- and BRWR-peel-extract-treated rats were reported to have higher inhibition (44.32–71.83%) than those treated with BRYR pulp and BRWR pulp extracts (43.65–55.63%), when compared with normal control (NC). At that time, indomethacin was reported to have a 63.26% inhibition value. In addition, BRYR peel extract was reported to have the most significant anti-inflammatory effect after 1 h of carrageenan injection among all other extracts, which was sustained throughout the experiment. Moreover, it was also found that the anti-inflammatory potential of BRYR peel was superior to that of indomethacin during 3–4 h of observation.

According to study, paw edema is associated with increased vascular permeability, cellular infiltration and fluid leakage from sites of inflammation [4]. Paw diameter measurement is a tool used to assess the effectiveness of anti-inflammatory remedies [14]. A reduction in paw diameter indicates a decline in the release of inflammatory mediators (IL-6 and TNF-α), which is a vital indicator for anti-inflammatory potential of drugs or plant extracts [2]. In the present study, it is found that *B. rapa* extracts exert a dose-reliant anti-inflammatory potential by reducing the paw diameter of inflamed rats. The greater inhibition of carrageenan-induced paw edema by BRYP-extract-treated rats can be elucidated by the existence of more polyphenols. Several pharmacological effects of polyphenols have been documented. In addition, polyphenols are acknowledged to retain anti-inflammatory potential [9]. For example, gallic acid possesses anti-inflammatory properties that could be due to restraint of pro-inflammatory mediators (IL-6 and TNF-α) [34]. Many studies have also been reported on the diverse therapeutic potential, including the anti-inflammatory and immunomodulatory potential, of chlorogenic acid [35]. Kaempferol is a flavonoid and exhibits anti-inflammatory properties [36]. Besides abundance of these polyphenols, higher TPC and TFC values of peel extracts also authenticated their anti-inflammatory potential. Therefore, it was suggested that the anti-inflammatory potential of *B. rapa* probably associated with its phenolic compounds. The findings of the current study are comparable with results of the study investigated by Semwal et al. [2]. In which Complete Freund’s Adjuvant (CFA)-injection-induced rat paw edema was significantly reduced by the ethanol extract of *Brassica rapa* (dose 200 mg/kg) [2].

#### 2.4.1. Suppression of Biochemical Inflammatory Biomarkers

**Figure 5:**
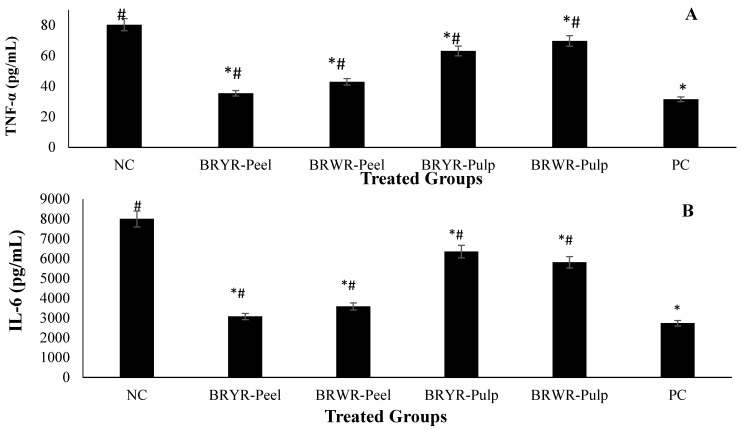
Effect of polyphenol-rich *B. rapa* extracts and indomethacin on suppression of TNF-α, and IL-6 in blood serum: (**A**) tumor necrosis factor (TNF)-α, and (**B**) IL-6. Results are presented as mean ± standard deviation (*n* = 6) and analyzed by one-way ANOVA (*p* ≤ 0.05). NC group: Normal control; BRYR-Peel group: *B. rapa* yellow root peel; BRWR-Peel group: *B. rapa* white root peel; BRYR-Pulp group: *B. rapa* yellow root pulp; BRWR-Pulp group: *B. rapa* white root pulp; PC: Positive control group. * symbolizes significant difference (*p* ≤ 0.05) as compared to NC. # symbolizes significant difference (*p* ≤ 0.05) as compared to PC.

The effect of polyphenol-rich BRYR peel, BRWR peel, BRYR pulp and BRWR pulp extracts and indomethacin on suppression of TNF-α, IL-6, CRP and RF in blood serum is given in Figure 5 and Table 4. The TNF-α level was higher in the normal control rat group (80.35 pg/mL). However, the TNF-α level was observed significantly (*p* ≤ 0.05) suppressed (ranged, 35.40–43.21 pg/mL) in *B. rapa* peel extracts compared to root pulp extracts (ranged, 63.64–70.92 pg/mL). Similarly, *B. rapa* peel extracts also showed lower concentration (3070–3580 pg/mL) IL-6 levels than those of root pulp extracts (5810–6350 pg/mL). Moreover, the normal control group showed a maximum level of CRP (6.91 mg/L) and RF (16.21 IU/mL) in the blood serum, showing the inflammation in the animals. Reduction in the raised levels of CRP and RF of treatment groups showed the effectiveness of the extracts. The maximum decrease in CRP (2.93 mg/L) and RF (10.93 IU/mL) levels was observed in the BRYR pulp group, which is analogous with the PC group. Overall, the most effective *B. rapa* extract, with significantly decreased levels of TNF-α, IL-6, CRP and RF in blood serum, was reported for BRYR peel. Thus, a significant anti-inflammatory effect of all the extracts, especially peel extracts, was observed in the acute inflammatory model by suppression of pro-inflammatory cytokines (TNF-α, IL-6, CRP, and RF).

Previous studies have shown that TNF-α is a significant element in the inflammatory response. It produces an intrinsic protective response by stimulating macrophages and releasing leukotrienes and kinins, which further activate the release of other inflammatory mediators [37]. Interleukin 6 (IL-6) is another important mediator which is released in the infected areas [38]. TNF-α and IL-6 appear to be major pro-inflammatory mediators tangled in the pathophysiology of inflammation. The decrease in these pro-inflammatory mediators in groups treated with BRYR peel, BRWR peel, BRYR pulp and BRWR pulp extracts demonstrated the immune-regulatory function of *B. rapa*. Previous research has shown a scientific correlation between TNF-α and IL-6 stimulation of CRP transcription and TNF-α and IL-6 involvement in inflammation [7]. The literature also reports that acute phase CRP reaction is the first acute protein that is described as a known sign of sensitive inflammatory biomarker and symptom of tissue deterioration [6]. It has been documented that CRP exhibits a significant contribution to the development of inflammation [3]. CRP and RF levels were elevated in inflamed rats, while standard drugs and all the extracts reduced these parameters, indicating a protective effect of the *B. rapa* extracts. In the present study, BRYR peel, BRWR peel, BRYR pulp and BRWR pulp extracts reduce the levels of these factors, thereby improving inflammation. These results have been confirmed by previous findings where *B. rapa* seed oil treatment regulates the level of TNF-α mRNA in rats with osteoporosis [39]. Furthermore, suppression of these pro-inflammatory cytokines is due to the existence of phenolic compounds [9]. Phenolic acids include sinapic acid, caffeic, cinnamic, and ferulic acids, identified in BRYR peel, BRWR peel, BRYR pulp and BRWR pulp extracts, and exhibit anti-inflammatory effects by decelerating TNF-α expression [40]. Flavonoids like catechin, quercetin, and kaempferol, also found in all *B. rapa* extracts, have the ability to inhibit IL-6 production as well as reduce CRP level and clinical signs of inflammation [10,11,36]. Therefore, these observations deliver a viable indication that this herbal product could be effective in the cure of inflammatory infections.

#### 2.4.2. Histopathology Study

**Figure 6:**
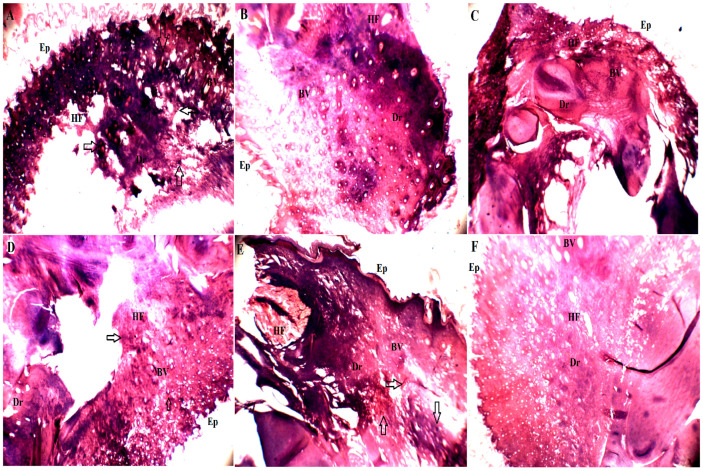
Effect of polyphenol-rich *B. rapa* extract on carrageenan-induced rat paw tissue by histopathological analysis. (**A**) Normal control group; (**B**) BRYR-Peel group (*B. rapa* yellow root peel); (**C**) BRWR-Peel group (*B. rapa* white root peel); (**D**) BRYR-Pulp group (*B. rapa* white root pulp); (**E**) BRWR-Pulp group (*B. rapa* white root pulp); (**F**) Positive control group (Indomethacin). Dr: Dermis; Ep: Epidermis; HF: Hair Follicles; BV: Blood Vessels. Arrows mark inflammation.

The histopathology analysis data of the NC, BRYR-peel, BRWR-peel, BRYR-pulp and BRWR-pulp PC groups is shown in Figure 6. It has been well characterized that neutrophil infiltration plays a significant character in carrageenan-induced paw inflammation. Microscopic examination of rats’ paws inflamed with carrageenan displayed connective tissue infiltration along with acute edema in the epidermis and dermis layers. Micrographs show that major edema and neutrophils were observed in normal control rats along with the deterioration of collagen tissue and high leukocyte infiltration in the dermis layer compared with the treated groups. Less inflammation was observed in the treated BRYR-Pulp and BRWR-Pulp groups than in the NC group. However, the BRYR-Peel and BRWR-Peel groups, and the PC (indomethacin-treated rat) group did not display any kind of inflammation or neutrophil cells. This means that the PC group, as well as the BRYR-Peel and BRWR-Peel groups, showed decreased leukocyte and collagen degradation than the BRYR-Pulp and BRWR-Pulp groups. Therefore, the histopathology study revealed that *B. rapa* extracts diminished leukocyte and collagen deterioration and improved vascularity by reducing inflammation. According to Li et al. [4], rats treated with alkaloid leaf extract of *E. cuneatum* managed to reduce infiltration of collagen and leukocyte tissue interruption in a dose-dependent manner, and these outcomes mirrored our current results.

### 2.5. Analgesic Activity

**Figure 7:**
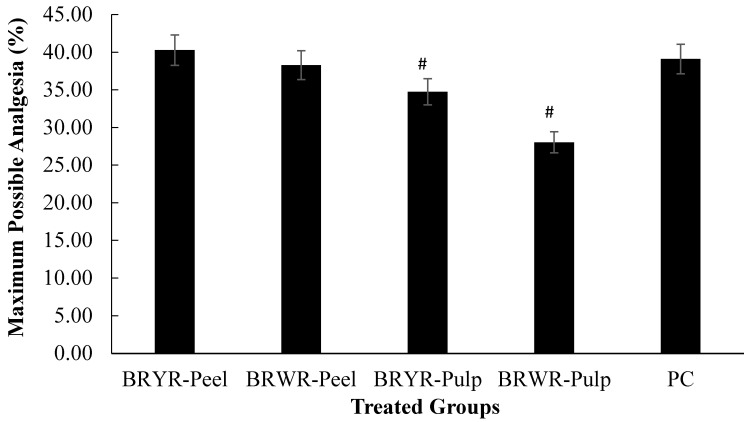
Effect of polyphenol-rich *B. rapa* extracts on analgesic activity as maximum possible analgesia (MPA) compared to indomethacin. NC group: Normal control; BRYR-Peel group: *B. rapa* yellow root peel; BRWR-Peel group: *B. rapa* white root peel; BRYR-Pulp group: *B. rapa* yellow root pulp; BRWR-Pulp group: *B. rapa* white root pulp; PC: Positive control group. # symbolizes significant difference (*p* ≤ 0.05) as compared to PC.

Table 5 represents the effect of BRYR peel, BRWR peel, BRYR pulp and BRWR pulp extracts and indomethacin on analgesic activity. The hot plate method was used to assess thermal stimulation in the skin and measures the time for the rat to jump or lick to analyze its potential effects [41]. Administration of *B. rapa* extracts (200 mg/kg) triggered an important stimulatory effect in the rat by elongating the latency time. At 90 min after the administration of the dose, BRYR peel and BRWR peel extracts (200 mg/kg) achieved maximum thermal stimulation latency. However, the PC group observed a maximum delay in heat stimulation at 60 min (min). BRYR pulp and BRWR pulp extracts were also observed with maximum thermal stimulation at 90 min after dose administration, compared to the control group. Figure 7 demonstrates the analgesic potential of indomethacin- and extract-treated groups as maximum possible analgesia (MPA). The MPA of the BRYR-peel group was found to be 40.28%, which is better than that of the PC group (39.10%). The BRWR-Peel, BRYR-Pulp and BRWR-Pulp groups displayed a significant (*p* ≤ 0.05) reduction in MPA. Overall, the BRYR-Peel group displayed an outstanding analgesic potential at 90 min (min) and so BRYR-Peel was found to be effective in relieving pain. According to the results of the present investigation, the analgesic potential of these BRYR peel, BRWR peel, BRYR pulp and BRWR pulp extracts might be exerted by the central nervous system [42]. A study on polyphenol-rich plants found that the plant’s polyphenols can relieve pain [11,43]. Another study reported the analgesic effect of phenolic and flavonoids compounds [43]. Moreover, RP-HPLC characterization of peel and pulp extracts of *B. rapa* varieties revealed the presence of gallic acid, quercetin and ferulic acid, which have exhibited strong analgesic potential in vivo studies [34,44,45]. These results are confirmed by previous findings where treatment with an alcoholic extract of *B. rapa* root provided significant pain relief in a dose-reliant manner [19].

## 3. Materials and Methods

### 3.1. Collection, Pretreatment and Storage of Samples

Two varieties of *B. rapa* (yellow and white) were collected from the controlled agricultural fields of the Ayyub Agriculture Research Institute, Jhang Road, Faisalabad, Pakistan. Samples were validated and verified (voucher no. 248-bot-21 and 249-bot-21) by the Taxonomist, Botany Department, Government College University Faisalabad. The samples were rinsed thoroughly with distilled water. The peel and root pulp were separated, sliced, and shaded to dry at room temperature, and ground into semi-powder (mesh size 80) with an electric blender (AG-640, ANEX, Karachi, Pakistan). The samples were then transferred to sealed plastic packets and kept at 4 °C.

### 3.2. Chemical and Reagents

All reference and standard chemicals including gallic acid, ferulic acid, *p*-hydroxybenzoic acid, chlorogenic acid, syringic acid, vanillic acid, sinapic acid, *p*-coumaric acid, salicylic acid, cinnamic acid, benzoic acid, rutin, kaempferol, catechin, quercetin, 2,2-diphenyl-1-picrylhydrazyl radical (DPPH•), carrageenan, Folin-Ciocalteu reagent and butylated hydroxyanisole (BHA) were acquired from Sigma-Aldrich (St Louis, MO, USA). ELISA kits from Elabscience and all other chemicals and reagents used in this study, such as ferric chloride, trichloroacetic acid, sodium hydrogen phosphate, sodium dihydrogen phosphate and methanol, were of analytical grade unless otherwise specified and acquired from Merck Co. (Darmstadt, Germany).

### 3.3. Extract Preparation

*Brassica rapa* yellow root (BRYR) peel, *Brassica rapa* white root (BRWR) peel, *Brassica rapa* yellow root (BRYR) pulp, and *Brassica rapa* white root (BRWR) pulp extracts were prepared by a reported method [12]. Briefly, ground root peel and root pulp samples (50 g per sample) were extracted using methanol (300 mL) by a soxhlet extractor (500 mL capacity) for 6–8 h. The extracts were filtered with Whatman filter paper no. 1 and concentrated by a rotary evaporator (EYELA, SB-651, Rikakikai Co., Ltd., Tokyo, Japan) at reduced pressure. The concentrated and desiccated extracts were weighed, and the following formula was used for estimation of yield.

The extracts were kept in a freezer (−4 °C) up until used for further studies.

$$\mathrm{Yield}\left(\frac{g}{100\;g}\right)=\frac{\mathrm{Dry}\;\mathrm{extract}\;\mathrm{weight}}{\mathrm{Dry}\;\mathrm{plant}\;\mathrm{material}\;\mathrm{weight}}\times 100$$

### 3.4. Quantification of Phenolic and Flavonoids Compounds by RP-HPLC

#### 3.4.1. Sample Preparation

For the quantitative and qualitative investigation of phenolic and flavonoid compounds from BRYR peel, BRWR peel, BRYR pulp and BRWR pulp extracts, a previously reported method was followed [12]. Briefly, 100 mg extract was dissolved in methanol (10 mg/mL), filtered through a 45-micron (um) nylon syringe filter, and kept at 4 °C. Fresh stock solutions of standards were made by dissolving authentic compounds in methanol (10 mg/mL). The standard solutions were diluted with methanol for further processing to obtain the requisite concentration (0.4–10 mg/mL). Standard concentration versus the area of peak was plotted to draw a calibration curve of each standard.

#### 3.4.2. Chromatographic Conditions

The Agilent 1260 infinity HPLC system (Agilent, Santa Clara, CA, USA) with a C-18 column (150 × 4.6 mm internal diameter, 2.7 micrometer (µm) particle size) equipped with a gradient binary pump system (G7112B), 1260 autosampler (G7129A) and multiwavelength detector (G7165A) was used. HPLC analysis data is given as a Supplementary Materials. The non-linear gradient with methanol was acetonitrile (30:70 as solvent A) and 0.1% acetic acid in distilled water as solvent B. The following gradient program was developed: 10% A from 0 to 5 min; 10–30% A from 5 to 25 min; 30–40% A from 25 to 40 min; 40–90% A from 40 to 60 min and kept at 90% A from 60–65 min. The MWD detector settings were 250, 270, 290, 310, 330, 350, 370 nm 1.2 nm resolution and 10 points/s sampling rate. Qualitative analysis was performed using the matching of retention times with the authentic standards and spiking of standards in the samples, while for quantitative analysis, the standard addition method was applied.

### 3.5. In Vitro Antioxidant Potential

#### 3.5.1. Total Phenolic Contents

The total phenolic contents (TPC) of BRYR peel, BRWR peel, BRYR pulp and BRWR pulp extracts were estimated by the Folin–Ciocalteu reagent as reported by Singleton et al. [46]. Gallic acid was used as standard (0.1–0.8 mg/mL) to prepare the standard curve (y = 0.9478x + 0.1097, R^2^ = 0.9965), and the findings were reported as mg/g of dried material as gallic acid equivalent.

#### 3.5.2. Total Flavonoid Contents

The total flavonoid contents (TPC) of BRYR peel, BRWR peel, BRYR pulp and BRWR pulp extracts were calculated as performed by Zhishen et al. [47]. The quercetin was used as standard (0.1–0.8 mg/mL) to prepare the standard curve (y = 0.7159x + 0.0342, R^2^ = 0.9973), and the findings were reported as mg/g of dried material, as quercetin equivalent.

#### 3.5.3. DPPH Radical Scavenging Assay

The antioxidant activity of BRYR peel, BRWR peel, BRYR pulp and BRWR pulp extracts, in term of potential to neutralized 2,2-diphenyl-1-picrylhydrazyl free radicals (DPPH), was examined by a published method [30]. The extract (10 µg/mL) was mixed in methanol with the same amount of DPPH solution (90 μmol/L in methanol). After incubating the solution for 30 min at ambient temperature (30 °C), the absorbance was noted at a wavelength of 517 nm. Butylated hydroxyanisole (BHA) was used as a positive standard. Radical scavenging percent concentrations were determined by the mean of the following formula:

$$\mathrm{Radical}\;\mathrm{Scavenging}\left(\%\right)=\frac{\mathrm{Absorbance}\;\mathrm{of}\;\mathrm{DPPH}\;\mathrm{solution}\;-\;\mathrm{Absorbance}\;\mathrm{of}\;\mathrm{sample}\;\mathrm{solution}}{\mathrm{Absorbance}\;\mathrm{of}\;\mathrm{DPPH}\;\mathrm{solution}}\times \;100$$

The 50% inhibition (IC_50_) of extract concentration was determined from the graph plotting extract concentration against radical scavenge percentage.

#### 3.5.4. Reducing Power

The procedure reported by Hussain et al. [12] was used to determine the reducing power of BRYR peel, BRWR peel, BRYR pulp and BRWR pulp extracts. Different concentrations (2–10 mg/mL) of extracts were used to determine the reducing potential. Absorbance was taken at 700 nm of wavelength nm by spectrophotometer (Lambda 25 UV/VIS, L600000B, Perkin Elmer Singapore, Singapore).

### 3.6. In Vivo Anti-Inflammatory and Analgesic Potentials

Healthy male Wistar Kyoto (WKY) rats (140–160 g) were purchased from the Animal House, Faculty of Pharmaceutical Sciences, Government College University Faisalabad. Before starting the experimental work, these animals were kept for 5 days in an animal house for acclimatization. The animal house maintained all the standard requirements, such as an ambient temperature (25 ± 3 °C), 12 h dark/light cycle and humidity range (30–70%), which were essential to the rats’ survival. The rats were fed normal rat chow and water ad libitum. One week after acclimatization, the rats were randomly distributed in different cages with six rats per group/cage. All animal-based experimental work was conducted with the Review Board approval at Government College University Faisalabad, Pakistan with an ethical permit, GCUF/ERC/26.

#### 3.6.1. Acute Inflammatory Model

##### Animal Grouping

For evaluating the anti-inflammatory potentials of BRYR peel, BRWR peel, BRYR pulp and BRWR pulp extracts, animals were divided into following groups, with 6 rats in each group: Normal Control (NC) group (received only standard pellet diet and normal saline orally), BRYR-peel group (received BRYR peel extract (200 mg/kg) along with pellet diet and normal saline orally), BRWR-Peel group (received BRWR peel extract (200 mg/kg) along with pellet diet and normal saline orally), BRYR-Pulp group (received BRYR pulp extract (200 mg/kg) along with pellet diet and normal saline orally), BRWR-Pulp group (received BRWR pulp extract (200 mg/kg) along with pellet diet and normal saline orally), Positive Control (PC) group (received Indomethacin (10 mg/kg) along with pellet diet and normal saline orally).

##### Study Design

To investigate acute inflammation, a carrageenan-induced paw edema model was performed using the described method [14]. The experimental groups (BRYR-Peel, BRWR-Peel, BRYR-Pulp and BRWR-Pulp) were treated with an extract dose of 200 mg/kg/day for 14 days, whereas the positive control group was given an indomethacin dose of 10 mg/kg only on the 14th day. On the 14th day, each rat was injected with 0.1 mL of 1% of freshly prepared carrageenan to induce inflammation below the plantar fascia of the right hind paw. A digital vernier caliper (Ugo Basile, model 7140, Gemonio, Italy) was used to measure paw diameter (PV_օ_) before and 1, 2, 3, 4 and 5 h after carrageenan injection (PV_t_) [48]. Edema inhibition in the treated groups vs. the control group was calculated using the following equation:

$$\%\;\mathrm{Inhibition}\;\mathrm{of}\;\mathrm{Inflammation}=\frac{{(PV_{t}\;-\;PV_{\circ })}_{\mathrm{control}}\;-\;{(PV_{t}-\;PV_{\circ })}_{\mathrm{treated}}}{{(PV_{t}-\;PV_{\circ })}_{\mathrm{control}}}\;\times \;100$$

where PV_t_ represents the diameter of inflammation in the rat treated after carrageenan and PV_օ_ represents the diameter of inflammation in the rat treated before carrageenan.

##### Suppression of Biochemical Inflammatory Biomarkers 

After experiment completion, rats of each group were euthanized by cervical decapitation. Blood samples and paw tissues were collected. Blood was collected in a vacuum vessel containing no anticoagulant, centrifuged it at 3000 rpm for 4 min (min), and a serum sample was obtained. Further, serum samples were analyzed by an automatic hemocytometer to examine suppression of biochemical biomarkers C-reactive protein (CRP) and rheumatoid factor (RF). The ELISA (Enzyme-Linked Immunosorbent Assay) was also performed using a kit protocol (Elabscience, E-EL-R0019, and E-EL-R0015 catalog numbers) to calculate tumor necrosis factor (TNF-α) and interleukin-6 (IL-6) concentration in blood serum [49].

##### Histopathology Study

For the histopathology study, each paw tissue sample was immersed in neutral formalin solution (10%), retained in paraffin wax, weighed, and stained by hematoxylin-eosin. Histopathological changes in the paw tissue were photographed under an optical microscope [4].

#### 3.6.2. Analgesic Activity

##### Animal Grouping

For evaluating analgesic activity of BRYR peel, BRWR peel, BRYR pulp and BRWR pulp extracts, animals were divided into following groups with 6 rats in each group: Normal Control (NC) group (received only standard pellet diet and normal saline orally), BRYR-Peel group (received BRYR peel extract (200 mg/kg) along with pellet diet and normal saline orally), BRWR-Peel group (received BRWR peel extract (200 mg/kg) along with pellet diet and normal saline orally), BRYR-Pulp group (received BRYR pulp extract (200 mg/kg) along with pellet diet and normal saline orally), BRWR-Pulp group (received BRWR pulp extract (200 mg/kg) along with pellet diet and normal saline orally), Positive Control (PC) group (received Indomethacin (10 mg/kg) along with pellet diet and normal saline orally).

##### Study Design

Analgesic potential was estimated by the hot plate process as described by Fan et al. [41] with slight modifications. The experimental groups (BRYR-Peel, BRWR-Peel, BRYR-Pulp and BRWR-Pulp) were given an extract dose of 200 mg/kg for 7 days, whereas the positive control group was treated with indomethacin 10 mg/kg. On the 7th day, analgesic activity was performed on all experimental group rats after 1 h of administration of extracts, and indomethacin. Each rat of all groups was placed on a hot plate in a glass beaker at 55 ± 0.5 °C. The reaction time of the rat against pain stimuli, and distress behavior including jumping and paw licking, was monitored. The latency time period or duration of the pain response was recorded by stopwatch in seconds. Before dosing, the first reading was observed at 0 time, while the remaining readings were observed at intervals of 30, 60, 90 and 120 minutes (min) after the extracts and drug intake. The cutoff time to prevent any injury was fixed at 12 seconds (s). This was considered as a control for pain response time. Maximum possible analgesia (MPA) was determined by the following formula:

$$\mathrm{MPA}\;\left(\%\right)=\frac{\mathrm{Response}\;\mathrm{time}\;\mathrm{for}\;\mathrm{treatment}\;-\;\mathrm{Response}\;\mathrm{time}\;\mathrm{for}\;\mathrm{saline}}{12\;-\;\mathrm{Response}\;\mathrm{time}\;\mathrm{for}\;\mathrm{saline}}\;\times \;100$$

### 3.7. Statistical Analysis

A Q-test was applied on each rat of each group to find the outlier values for both activities, but no outlier values were noticed. The sample of each variety of *B. rapa* was analyzed separately in triplicate. The data were represented as mean ± standard deviation (SD). One-way analysis of variance (ANOVA) was applied with the statistical package (Minitab, Version 17, Minitab Inc., State College, PA, USA) and the Tukey test was applied to compare the differences between mean values; differences were considered statistical significant if the probability value (*p*) was ≤0.05.

## 4. Conclusions

It can be concluded that the root peel extracts of both varieties of *B. rapa* exhibited more antioxidant potential than the root pulp extracts due to the presence of more polyphenols, and the best antioxidant activity was recorded with BRYP extract. Ten phenolic acids and four flavonoids were detected from the *B. rapa* extracts. Gallic acid and chlorogenic acid were the phenolic acids present in abundance in all extracts, whereas catechin was reported in the highest concentration in BRYR extract. A reduced rate of inflammation and suppressed level of pro-inflammatory biomarkers were observed in the BRYR-Peel and BRWR-Peel groups as compared with the BRYR-Pulp and BRWR-Pulp groups. TNF-α, IL-6, CRP and RF levels were lowest in the BRYR-Peel group, followed by the BRWR-Peel and root-pulp-extract-treated rat groups. Additionally, a decrease in leukocyte infiltration and collagen degradation were observed in rats treated with root-peel extracts rather than root pulp extracts by histopathology study. Analgesic activity results also showed that the rats given the peel extracts experienced less pain compared with the rats given the root pulp extracts. The plant extracts follow an extract-dependent suppression of TNF-α, IL-6, CRP and RF levels to reduce the development of inflammation and pain. BRYP at a dose of 200 mg/kg is the best anti-inflammatory extract with the greatest suppression potential of pro-inflammatory cytokines (TNF-α, IL-6, CRP and RF) along with antioxidant and analgesic activities. Further study can be planned to isolate specific compounds from *B. rapa* responsible for the suppression of pro-inflammatory cytokines (TNF-α, IL-6, CRP and RF), free radicals and pain-causing agents. The transduction pathways to treat acute and chronic inflammation and various pains should also be tested.

## Figures and Tables

**Table 1:** Extract yield and antioxidant activity of BRYR peel, BRWR peel, BRYR pulp and BRWR pulp methanol extracts.

Extracts	Yield (g/100 g)	TPC (mg GAE/g)	TFC (mg QE/g)	DPPH, IC_50_ (µg/mL)
BRYR peel	8.25 ± 0.41 ^b^	5.01 ± 0.25 ^d^	3.95 ± 0.20 ^d^	3.85 ± 0.19 ^b^
BRWR peel	6.72 ± 0.34 ^a^	3.21 ± 0.16 ^c^	2.28 ± 0.11 ^c^	4.64 ± 0.23 ^c^
BRYR pulp	13.35 ± 0.67 ^d^	2.16 ± 0.11 ^b^	1.30 ± 0.07 ^b^	5.08 ± 0.25 ^c^
BRWR pulp	10.63 ± 0.53 ^c^	1.21 ± 0.06 ^a^	0.90 ± 0.08 ^a^	5.76 ± 0.29 ^d^
BHA	--------	--------	--------	1.24 ± 0.06 ^a^

Values are mean ± standard deviation of triplicate experiments. Superscripted different letters indicate the significant difference (*p* ≤ 0.05) between different extracts. BRYR: *B. rapa* yellow root; BRWR: *B. rapa* white root; BHA: butylated hydroxyanisole; TPC: mg gallic acid equivalent (GAE)/g of dry material; TFC: mg quercetin equivalent (QE)/g of dry material.

**Table 2:** Composition of phenolic acids and flavonoids from methanol extracts of BRYR peel, BRWR peel, BRYR pulp and BRWR pulp by RP-HPLC.

Peak No.	Compounds	Concentration (mg/100 g of Dry Plant Material)			
		BRYR Peel	BRWR Peel	BRYR Pulp	BRWR Pulp
**1**	Gallic acid	642.3 ± 32.1 ^a^	174.6 ± 8.7 ^d^	228.1 ± 11.4 ^c^	330.5 ± 16.5 ^b^
**2**	*p*-hydroxyl benzoic acid	43.25 ± 2.16 ^bc^	26.20 ± 1.31 ^c^	46.51 ± 2.33 ^b^	65.33 ± 3.27 ^a^
**3**	Chlorogenic acid	84.3 ± 4.2 ^d^	196.3 ± 9.8 ^c^	237.2 ± 11.9 ^b^	267.1 ± 13.3 ^a^
**4**	Vanillic acid	-	-	9.31 ± 0.47	-
**5**	Syringic acid	104.0 ± 5.2	-	-	-
**6**	*p*-coumaric acid	186.3 ± 9.3 ^b^	108.4 ± 5.4 ^c^	-	207.3 ± 10.3 ^a^
**7**	Sinapic acid	1.43 ± 0.07 ^c^	0.41 ± 0.02 ^c^	19.03 ± 0.95 ^a^	3.43 ± 0.17 ^b^
**8**	Ferulic acid	18.60 ± 0.93 ^a^	3.13 ± 0.16 ^b^	0.99 ± 0.05 ^bc^	0.39 ± 0.02 ^c^
**9**	Rutin	-	-	4.63 ± 0.23	-
**10**	Cinnamic acid	78.77 ± 3.94 ^a^	21.24 ± 1.06 ^b^	8.15 ± 0.41 ^b^	3.23 ± 0.16 ^b^
**11**	Benzoic acid	1.10 ± 0.06 ^b^	3.05 ± 0.15 ^a^	-	-
**12**	Catechin	358.5 ± 17.9 ^a^	138.0 ± 6.9 ^c^	34.45 ± 1.72 ^d^	196.8 ± 9.83 ^b^
**13**	Quercetin	7.24 ± 0.36 ^b^	11.38 ± 0.57 ^a^	5.74 ± 0.29 ^b^	7.24 ± 0.36 ^b^
**14**	Kaempferol	16.39 ± 0.82 ^c^	34.04 ± 1.72 ^a^	31.5 ± 1.57 ^b^	-

Superscripted different letters indicate the significant difference (*p* ≤ 0.05) between different extracts. BRYR: *B. rapa* yellow root; BRWR: *B. rapa* white root; BHA: (Butylated hydroxyanisole).

**Table 3:** Effect of polyphenol-rich *B. rapa* extracts and indomethacin on % inhibition of inflammation in carrageenan-induced paw edema in rats.

Groups	% Inhibition of Inflammation				
	1 h	2 h	3 h	4 h	5 h
BRYR-Peel	57.82 ± 2.89 ^#^	59.83 ± 2.99 ^#^	71.83 ± 5.59	70.86 ± 3.54	68.49 ± 3.42 ^#^
BRWR-Peel	41.69 ± 2.08 ^#^	47.84 ± 2.39 ^#^	44.32 ± 2.22 ^#^	54.17 ± 1.71 ^#^	48.37 ± 1.42 ^#^
BRYR-Pulp	35.47 ± 1.77 ^#^	38.83 ± 1.94 ^#^	55.63 ± 2.78 ^#^	48.41 ± 2.42 ^#^	37.66 ± 1.88 ^#^
BRWR-Pulp	30.57 ± 1.53 ^#^	35.60 ± 1.78 ^#^	43.65 ± 2.18 ^#^	37.93 ± 1.90 ^#^	30.04 ± 1.50 ^#^
PC	73.93 ± 3.70	68.30 ± 3.42	63.26 ± 3.16	66.37 ± 3.32	80.57 ± 4.03

Results inhibition percentage ± standard deviation (*n* = 6) when compared with the positive control group (Indomethacin). BRYR-Peel: *B. rapa* yellow root peel, BRWR-Peel: *B. rapa* white root peel, BRYR-Pulp: *B. rapa* yellow root pulp, BRWR-Pulp: *B. rapa* white root pulp, PC: Positive control. ^#^ symbolizes significant difference (*p* ≤ 0.05) as compared to PC.

**Table 4:** Effect of polyphenol-rich *B. rapa* extracts and indomethacin on suppression of CRP and RF in blood serum of rat groups.

Groups	CRP (mg/L)	RF (IU/mL)
NC	6.91 ± 0.35 ^#^	16.21 ± 0.81 ^#^
BRYR-Peel	2.93 ± 0.15 *	10.93 ± 0.55 *
BRWR-Peel	3.96 ± 0.20 *	12.36 ± 0.62 *
BRYR-Pulp	4.52 ± 0.23 *^#^	13.97 ± 0.70 *^#^
BRWR-Pulp	5.61 ± 0.28 *^#^	14.32 ± 0.72 *^#^
PC	3.14 ± 0.16 *	10.80 ± 0.61 *

Statistical significance according to ANOVA (*p* ≤ 0.05). NC: Normal control (NC); BRYR-Peel: *B. rapa* yellow root peel; BRWR-Peel: *B. rapa* white root peel; BRYR-Pulp: *B. rapa* yellow root pulp; BRWR-Pulp: *B. rapa* white root pulp; PC: Positive control. * symbolizes significant difference (*p* ≤ 0.05) as compared to NC. ^#^ symbolizes significant difference (*p* ≤ 0.05) as compared to PC.

**Table 5:** Effect of polyphenol-rich *B. rapa* extracts and indomethacin on analgesic activity.

Groups	Reaction Time (min)				
	Start Time	30	60	90	120
NC	3.50 ± 0.20	3.52 ± 0.21 ^#^	3.53 ± 0.22 ^#^	3.51 ± 0.20 ^#^	3.55 ± 0.22 ^#^
BRYR-Peel	3.60 ± 0.27	6.19 ± 0.31 *^#^	6.53 ± 0.33 *	6.93 ± 0.35 *	6.29 ± 0.31 *^#^
BRWR-Peel	3.58 ± 0.23	5.81 ± 0.29 *^#^	6.17 ± 0.31 *^#^	6.76 ± 0.34 *	6.14 ± 0.31 *
BRYR-Pulp	3.57 ± 0.22	5.63 ± 0.41 *	6.11 ± 0.31 *^#^	6.46 ± 0.32 *	5.93 ± 0.30 *
BRWR-Pulp	3.55 ± 0.21	5.43 ± 0.28 *	5.47 ± 0.27 *^#^	5.89 ± 0.29 *^#^	5.43 ± 0.27 *
PC	3.52 ± 0.25	6.41 ± 0.32 *	6.91 ± 0.35 *	6.83 ± 0.34 *	5.79 ± 0.29 *

Reaction time ± standard deviation (*n* = 6) when compared with the positive control group (Indomethacin). Statistical significance according to ANOVA (*p* ≤ 0.05). NC: Normal control (NC); BRYR-Peel: *B. rapa* yellow root peel; BRWR-Peel: *B. rapa* white root peel; BRYR-Pulp: *B. rapa* yellow root pulp; BRWR-Pulp: *B. rapa* white root pulp; PC: Positive control. * symbolizes significant difference (*p* ≤ 0.05) as compared to NC. ^#^ symbolizes significant difference (*p* ≤ 0.05) as compared to PC.

## Data Availability

All data included in this study are available upon request by contacting the corresponding authors.

## Supplementary Materials

The following supporting information can be downloaded at: https://www.mdpi.com/article/10.3390/molecules29010117/s1.
