# Supplementary material for: Inter-Varietal Variation in Phenolic Profile, Antioxidant, Anti-Inflammatory and Analgesic Activities of Two Brassica rapa Varieties: Influence on Pro-Inflammatory Mediators
Source: Molecules. 2023 Dec 24;29(1):117. doi: 10.3390/molecules29010117 (PMC10779636; doi:10.3390/molecules29010117)

Single Injection Report

Agilent Technologies

Central Hi-Tech Lab. Governmnet College University
Faisalabad

Project Name	HPLC-Data	Instrument Name	HPLC-1260
Sample Name	Turnip Phenolics sample 2	Sample Vial Number	P1-A2

Name	RT	Area	Peak Area Percent	Unit	Concentration []
	2.925	22.2191	2.63		
	2.929	36.5001	1.02		
	2.930	133.2810	1.68		
	2.962	119.0644	1.73		
	2.983	171.9104	2.51		
	3.320	146.7718	17.39		
	3.321	1523.0272	22.13		
1	3.325	1513.2587	19.04		
	3.325	1333.6959	19.47		
	3.327	376.9227	10.52		
	3.604	58.8307	6.97		
	3.605	528.6407	7.72		
	3.611	172.9207	4.82		
	3.638	726.1009	10.55		
2	3.643	763.4493	9.60		
	4.396	198.6113	2.90		
	4.399	16.4988	0.46		
	4.399	146.9968	2.14		
	4.400	11.3673	1.35		
3	4.402	120.3722	1.51		
	4.582	97.5334	1.42		
	4.599	120.8865	1.52		
	4.702	27.9570	0.41		
	6.088	118.3546	1.49		
	6.088	167.3613	2.43		
	6.751	30.9565	0.39		
	9.755	164.2925	2.40		
	12.854	116.8401	1.47		
4	13.240	34.3998	0.43		
	13.247	546.9751	7.95		
	13.247	754.1395	11.01		
	14.821	59.9551	0.75		

Name	RT	Area	Peak Area Percent	Unit	Concentration []
	15.873	165.5135	2.42		
	15.879	55.4794	0.81		
	15.881	55.1596	0.69		
	16.502	10.6793	0.16		
	18.540	102.8566	1.29		
	18.549	128.9732	1.87		
	18.559	85.7897	1.25		
	19.391	49.8447	5.91		
5	19.391	105.7375	1.33		
	19.392	98.2904	1.43		
	19.392	118.3366	3.30		
	19.393	42.6538	0.62		
	20.296	89.2865	1.12		
	20.316	49.3996	0.72		
	20.317	95.1772	1.38		
	21.426	39.1468	0.57		
	21.609	16.0385	0.45		
	21.902	19.4008	0.24		
	22.042	110.3417	1.61		
	22.115	54.2134	0.79		
	22.117	59.7387	1.67		
	22.136	24.8850	0.31		
	22.137	4.7149	0.56		
	22.375	16.8533	2.00		
	22.376	19.8144	0.29		
	22.377	47.5370	1.33		
	22.377	59.7552	0.75		
	22.381	10.2720	0.15		
	22.632	20.5210	0.57		
	22.636	32.6388	0.41		
	22.639	10.2812	0.15		
	22.912	15.7580	0.20		
	22.919	11.6081	0.17		
	23.598	115.8502	1.68		
	23.619	221.6989	3.24		
	23.620	278.0583	3.50		
6	23.622	435.1079	12.14		

Name	RT	Area	Peak Area Percent	Unit	Concentration []
	23.622	50.0512	5.93		
	23.919	75.3159	1.10		
	23.921	63.1750	0.92		
	24.330	241.2768	28.59		
	24.330	449.4698	12.54		
	24.331	489.9120	7.12		
8	24.331	559.6510	7.04		
	24.332	239.9399	3.50		
	24.968	114.4004	1.44		
	24.978	56.3837	0.82		
	24.980	48.7609	0.71		
	25.681	11.2048	0.31		
	25.682	31.0429	0.45		
	25.687	8.4054	0.11		
	26.244	10.3342	0.15		
	26.246	19.4810	0.28		
	26.440	57.3330	6.79		
	26.442	93.6389	2.61		
	26.459	151.4403	1.91		
	26.475	169.8309	2.47		
	26.548	122.9184	1.79		
	27.361	11.8333	0.15		
	27.597	75.9499	1.10		
	27.598	184.3508	2.69		
	27.607	9.4256	0.12		
	28.624	57.9838	6.87		
	28.625	744.7999	20.78		
	28.625	584.7813	7.36		
	28.626	458.3463	6.69		
	28.627	161.4568	2.35		
10	28.912	136.3301	1.71		
	28.913	156.7290	4.37		
	28.913	147.4925	2.15		
	28.915	63.3308	0.92		
	28.919	15.4120	1.83		
	29.265	18.3707	0.27		
	29.476	11.5480	0.17		

Name	RT	Area	Peak Area Percent	Unit	Concentration []
	29.516	13.3047	0.37		
	29.776	7.3538	0.11		
	29.778	7.3021	0.11		
	30.555	7.0697	0.20		
	30.641	70.8763	0.89		
	30.645	57.9676	0.85		
	30.646	49.2883	0.72		
	30.877	9.7219	0.27		
	31.158	20.0969	2.38		
	31.159	18.7377	0.27		
	31.159	124.6844	1.57		
	31.159	178.7638	4.99		
	31.161	71.8197	1.05		
	31.369	20.9004	0.31		
	31.369	78.5423	2.19		
	31.371	58.0389	0.73		
	31.375	41.2146	0.60		
	31.375	27.5510	3.26		
	31.588	6.9168	0.10		
	31.797	94.5137	1.38		
	31.798	61.2333	0.89		
	31.799	46.6347	0.59		
	31.818	37.9121	1.06		
11	32.850	277.1994	3.49		
	32.850	324.2721	9.05		
	32.851	176.8897	2.58		
	32.851	44.9837	5.33		
	32.853	71.8306	1.04		
	33.204	5.1271	0.07		
	33.206	10.8625	0.16		
	33.376	12.7960	0.36		
	33.727	13.9856	0.20		
	33.734	34.9446	0.51		
	33.736	33.5244	0.42		
	33.849	5.6627	0.16		
	34.018	14.7505	0.41		
	34.023	38.6791	0.49		

Single Injection Report

Agilent Technologies

Name	RT	Area	Peak Area Percent	Unit	Concentration []
	34.024	36.1906	0.53		
	34.025	18.7460	2.22		
	34.026	60.7849	0.88		
	34.318	15.5189	0.23		
	34.324	14.4432	0.21		
	34.539	10.8929	0.30		
	35.172	36.9400	0.54		
	35.176	17.2126	0.25		
	35.182	17.8546	0.22		
	35.187	29.6224	0.83		
	35.355	24.1289	0.67		
	36.655	241.0196	3.50		
	36.661	180.6851	2.64		
12	36.666	133.3065	1.68		
	36.682	53.8487	1.50		
	37.767	27.3345	0.40		
	37.969	16.7545	0.24		
	37.972	57.9535	0.73		
	38.904	10.3546	0.15		
	38.905	10.1969	0.13		
	38.905	16.7984	0.24		
	40.416	17.0191	0.21		
	42.106	66.1868	0.96		
	42.106	63.6778	0.93		
	42.107	32.7640	0.41		
	44.213	13.1638	0.37		
	44.214	42.1682	0.62		
	44.214	11.2608	0.16		
	46.177	17.2734	0.22		
	46.682	14.0096	0.39		
	46.812	33.5976	0.49		
	46.822	69.6798	0.88		
	47.649	74.2432	0.93		
	49.721	14.2765	0.21		
	50.051	21.0317	0.26		
	50.057	18.3012	0.27		
	51.982	81.5432	1.03		

Single Injection Report

Agilent Technologies

Name	RT	Area	Peak Area Percent	Unit	Concentration []
	52.644	87.0812	1.10		
	52.981	34.5677	0.43		
	53.195	22.8815	0.29		
	53.871	35.3354	0.52		
	53.872	11.0187	0.14		
	53.872	35.2579	0.51		
13	54.204	165.1501	2.08		
	54.207	234.2433	3.42		
	54.207	313.2586	4.55		
	54.544	9.2489	0.13		
14	54.812	607.3317	7.64		
	54.813	69.4235	1.01		
	54.813	65.0335	0.94		
	55.129	105.5268	1.33		
	55.854	9.3828	0.12		
	55.870	19.8900	0.29		
	55.871	26.3733	0.38		
	56.207	8.4312	0.11		
	56.210	31.4079	0.46		
	56.211	33.0158	0.48		
	56.696	111.8814	1.63		
	56.697	51.1440	0.64		
	56.697	151.5207	2.20		
	57.235	60.5596	0.76		
	57.507	13.9572	0.18		
	57.671	25.3004	0.32		
	58.174	33.2544	0.42		
	58.194	29.3119	0.43		
	58.195	31.2043	0.45		
	58.404	78.4923	0.99		
	58.404	147.4410	2.15		
	58.405	208.1412	3.02		
	59.333	11.5068	0.14		
	59.851	7.4903	0.09		
	60.002	12.7056	0.19		
	60.003	19.9202	0.29		
	60.136	19.4254	0.28		

Single Injection Report

Agilent Technologies

Name	RT	Area	Peak Area Percent	Unit	Concentration []
	60.137	17.3635	0.25		
	60.466	56.0915	0.82		
	60.466	83.4872	1.21		
	60.467	30.6823	0.39		
	60.923	31.7110	0.40		
	61.390	7.1724	0.10		
	61.398	12.1694	0.18		

Single Injection Report

Agilent Technologies

MWD1A,Sig=250,4 Ref=off

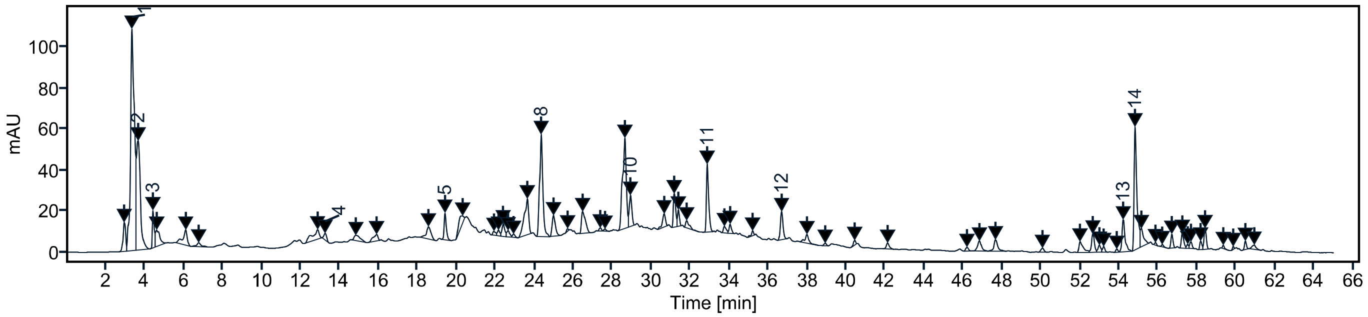

MWD1B,Sig=270,4 Ref=off

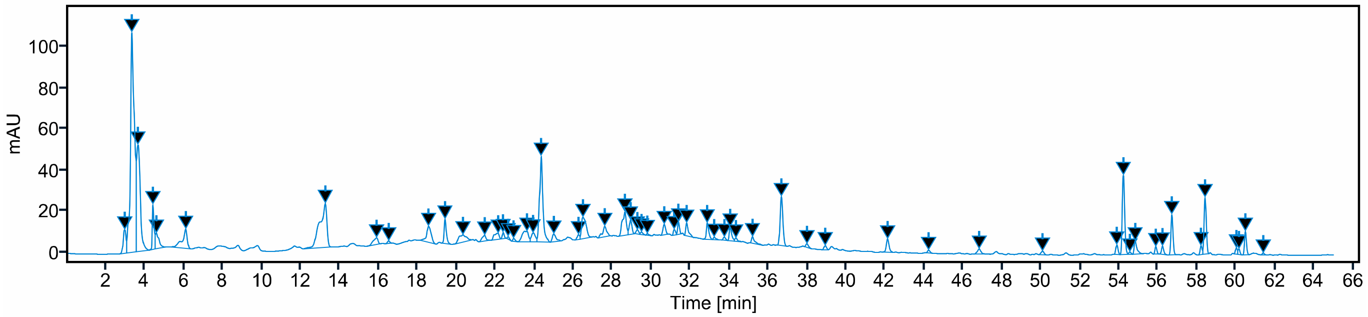

MWD1C,Sig=290,4 Ref=off

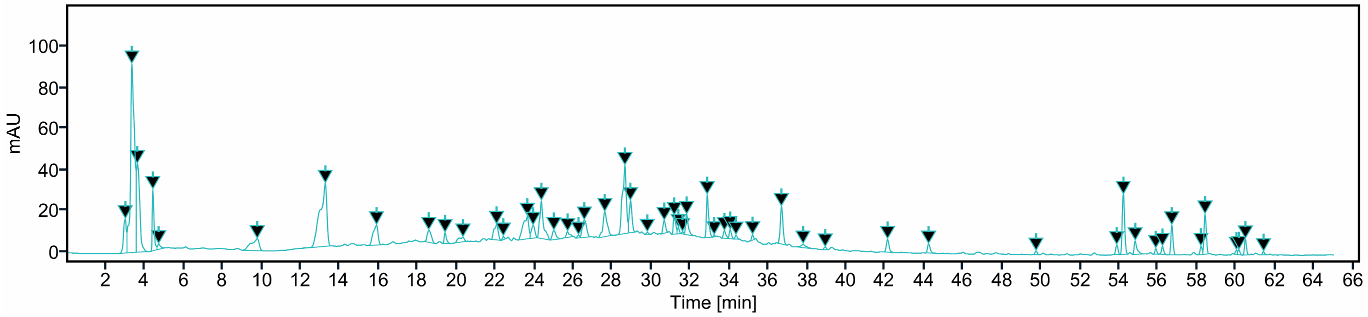

MWD1D,Sig=350,4 Ref=off

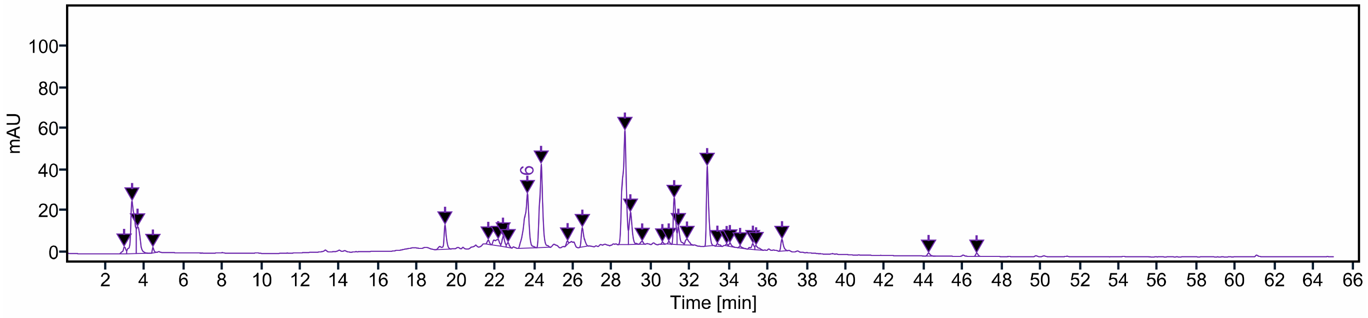

MWD1E,Sig=380,4 Ref=off

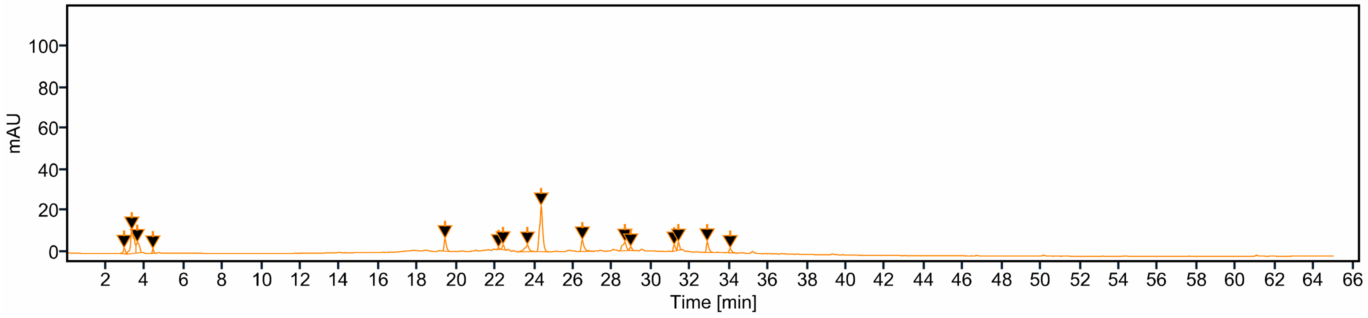

Single Injection Report

Agilent Technologies

Central Hi-Tech Lab. Governmnet College University
Faisalabad

Project Name	HPLC-Data	Instrument Name	HPLC-1260
Sample Name	Turnip Phenolics sample 1	Sample Vial Number	P1-A1

Name	RT	Area	Peak Area Percent	Unit	Concentration []
	0.080	10.1940	0.95		
	0.080	10.4170	2.56		
	0.086	34.1331	0.67		
	0.089	34.1243	0.67		
	0.091	55.2761	1.18		
	2.876	31.1574	7.65		
	2.883	300.8134	5.91		
	2.883	969.7897	18.93		
	2.885	250.7194	5.34		
	3.032	298.3085	5.86		
	3.186	2744.8635	53.92		
	3.186	770.4768	71.87		
	3.187	2115.9557	45.04		
	3.187	2260.0976	44.11		
	3.187	311.0624	76.38		
	3.932	21.8408	5.36		
	3.933	34.5940	3.23		
	3.935	171.1526	3.34		
	3.936	199.1467	3.91		
	3.937	244.7090	5.21		
	4.217	78.1056	1.52		
	4.255	196.0910	3.85		
	4.262	140.8696	3.00		
	9.106	82.2875	1.62		
	9.107	222.6588	4.35		
	11.341	69.4409	1.48		
	12.856	32.3772	0.69		
	12.861	208.0665	4.09		
	12.862	270.6974	5.28		
	15.673	83.2654	1.77		
	15.675	181.4576	3.54		
	15.677	63.9260	1.26		

Agilent Technologies

Name	RT	Area	Peak Area Percent	Unit	Concentrati on []
	18.375	8.2427	0.16		
	18.376	17.5304	0.37		
	19.299	14.7322	1.37		
	20.177	53.4880	1.14		
	20.187	53.5978	1.05		
	20.224	47.9138	0.94		
	20.467	12.5591	0.25		
	21.364	18.4506	0.36		
	21.365	19.2790	0.41		
	22.498	18.7812	0.37		
	23.321	9.3900	0.88		
	23.509	42.6589	0.84		
	23.535	35.0304	0.75		
6	23.565	11.0937	1.03		
	23.841	18.4826	0.39		
	23.849	34.6234	0.68		
	23.850	55.7617	1.09		
	24.287	54.9700	5.13		
	24.288	32.7606	8.04		
	24.288	51.3571	1.01		
	24.289	21.5337	0.42		
8	24.291	76.4002	1.63		
	24.969	9.3068	0.18		
	24.972	7.8337	0.17		
	24.977	15.2988	0.30		
	26.170	10.6269	0.21		
	26.174	15.3998	0.30		
	26.481	14.9058	0.29		
	26.852	22.8987	0.45		
	27.596	23.8179	0.46		
	27.600	11.5125	0.23		
	28.579	16.8539	0.33		
	28.579	56.6084	1.10		
	28.581	74.8881	6.99		
	28.581	56.9407	1.21		
10	28.864	20.6622	0.44		
	28.867	24.3343	0.47		

Single Injection Report

Agilent Technologies

Name	RT	Area	Peak Area Percent	Unit	Concentration []
	28.867	24.2501	2.26		
	30.592	68.7148	1.35		
	30.593	82.7966	1.76		
	30.594	81.7650	1.60		
	31.128	10.7777	0.23		
	31.129	15.7658	1.47		
	31.757	30.9725	0.66		
	31.761	113.1464	2.21		
	31.762	70.5095	1.39		
11	32.822	28.1255	0.60		
	32.824	37.7001	3.52		
	32.826	17.9218	0.35		
	32.834	11.9081	0.23		
	33.177	18.3064	0.36		
	33.178	16.4511	0.32		
	33.959	13.6397	0.29		
	34.285	12.5368	0.25		
	34.287	23.9668	0.47		
	36.605	233.8079	4.59		
	36.606	158.9857	3.10		
12	36.609	102.9582	2.19		
	36.636	13.9784	1.30		
	37.922	18.4959	0.39		
	38.856	19.2226	0.41		
	38.857	18.7824	0.37		
	38.858	29.8754	0.59		
	40.376	40.7609	0.87		
	42.065	49.4753	0.97		
	42.065	23.1185	0.49		
	42.065	52.1518	1.02		
	44.174	26.0713	0.51		
	46.134	15.5823	0.33		
	46.786	42.2692	0.90		
	47.604	57.3953	1.22		
	51.953	65.4231	1.39		
	52.613	23.0406	0.49		
	52.951	14.5731	0.31		

Single Injection Report

Name	RT	Area	Peak Area Percent	Unit	Concentration []
	53.168	8.4813	0.18		
13	54.173	54.2934	1.16		
	54.184	45.8614	0.90		
	54.185	61.0454	1.20		
14	54.792	683.8482	14.55		
	54.793	73.8918	1.44		
	54.793	65.6034	1.29		
	55.852	6.0928	0.12		
	55.852	8.1320	0.16		
	56.677	9.9203	0.21		
	56.678	19.8111	0.39		
	56.678	27.0131	0.53		
	58.143	27.4284	0.58		
	59.835	12.1948	0.26		
	60.918	14.8903	0.32		

Single Injection Report

Agilent Technologies

MWD1A,Sig=250,4 Ref=off

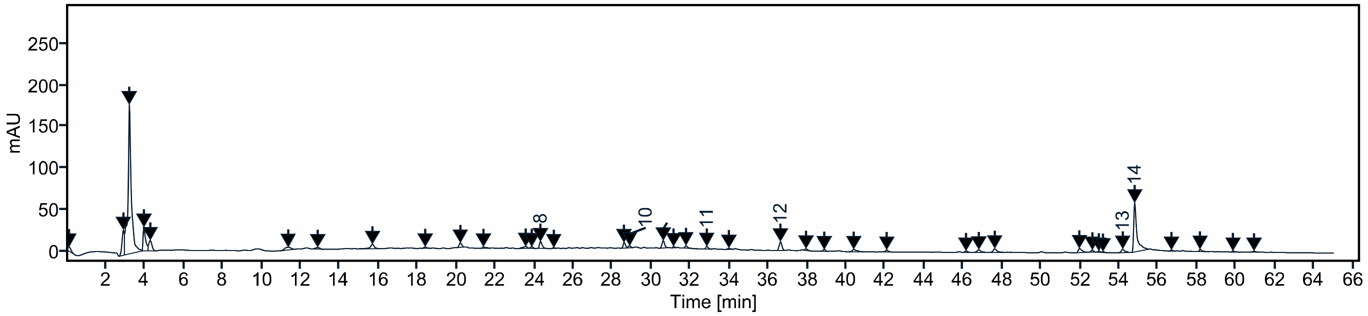

MWD1B,Sig=270,4 Ref=off

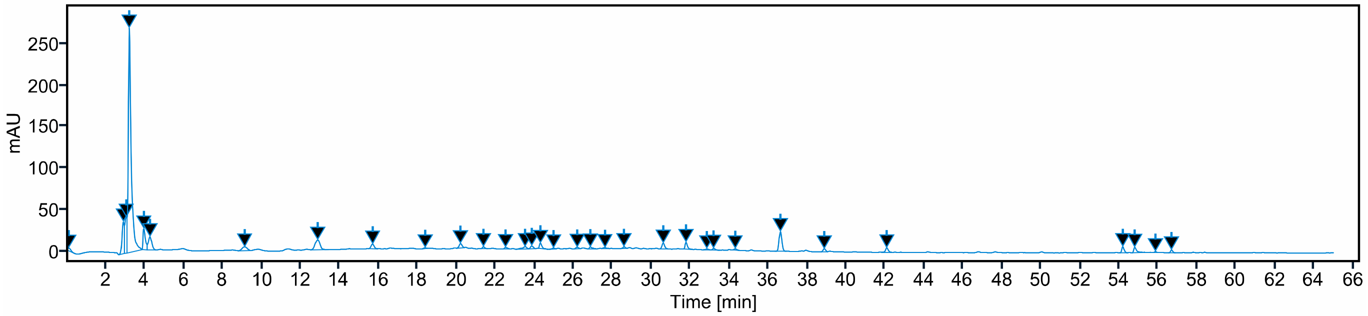

MWD1C,Sig=290,4 Ref=off

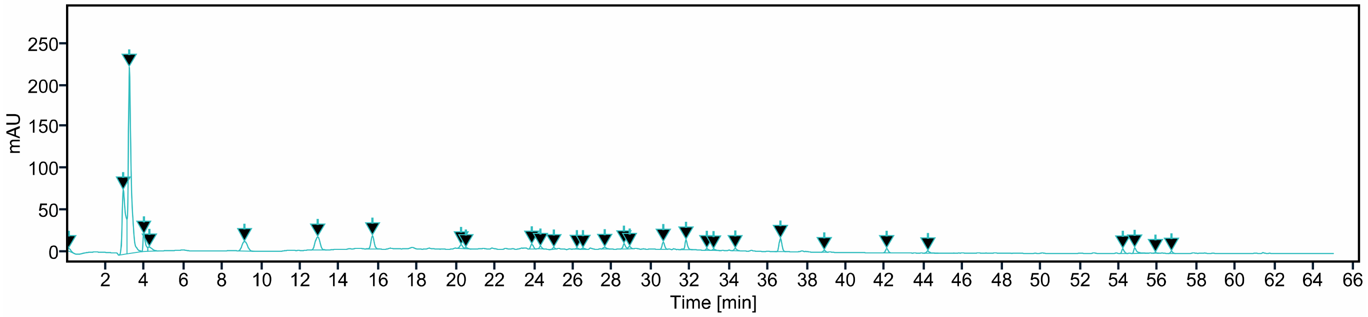

MWD1D,Sig=350,4 Ref=off

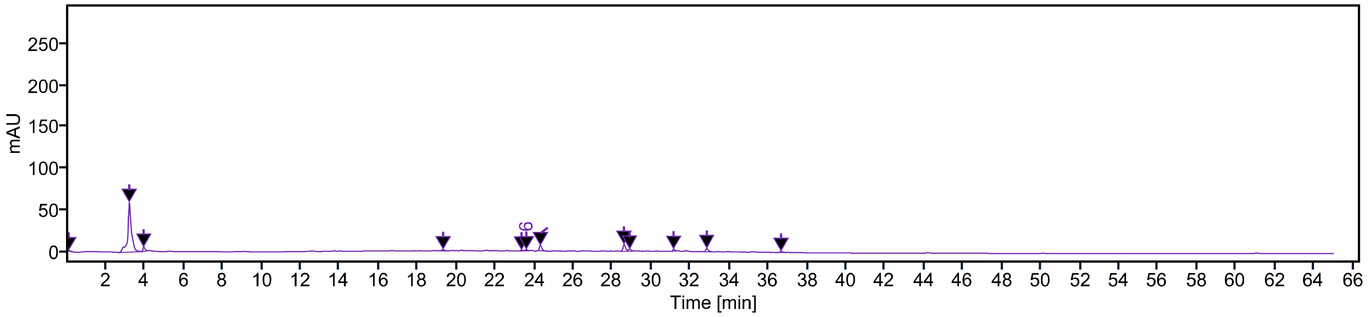

MWD1E,Sig=380,4 Ref=off

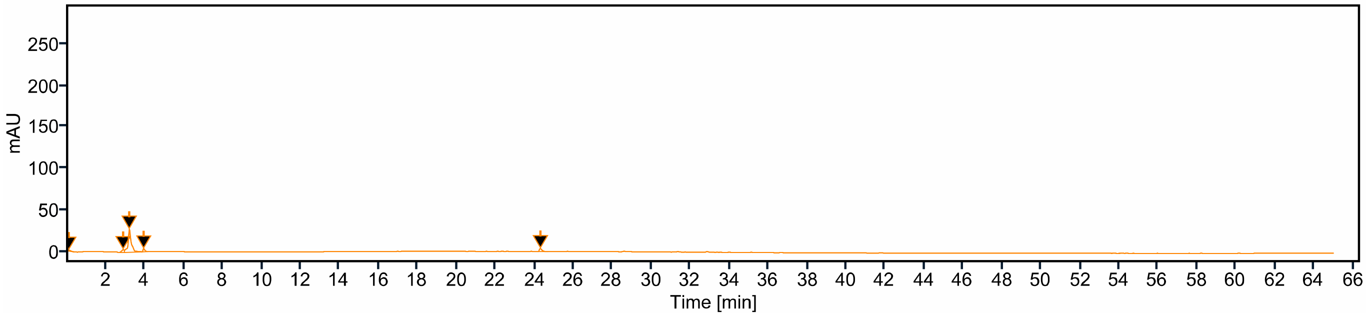

Single Injection Report

Agilent Technologies

Central Hi-Tech Lab. Governmnet College University
Faisalabad

Project Name	HPLC-Data	Instrument Name	HPLC-1260
Sample Name	Turnip Phenolics sample 3	Sample Vial Number	P1-A3

Name	RT	Area	Peak Area Percent	Unit	Concentration []
	2.933	18.4164	3.87		
	2.935	133.0012	2.96		
	3.005	14.2238	1.67		
	3.013	62.8326	1.17		
	3.024	100.0180	1.78		
	3.322	227.1821	47.75		
1	3.323	1201.9117	26.71		
	3.323	1251.0715	22.28		
	3.324	432.9164	50.77		
	3.325	1465.0216	27.25		
	3.518	296.8607	5.29		
	3.522	126.6259	14.85		
	3.524	74.0984	15.57		
	3.526	313.3724	5.83		
	3.529	308.5757	6.86		
	3.615	590.4006	10.51		
	3.624	247.2091	28.99		
	3.624	142.2774	29.90		
	3.626	825.4728	15.35		
2	3.628	801.3194	17.80		
	4.406	141.3995	2.52		
	4.408	20.6891	2.43		
	4.409	13.8412	2.91		
	4.410	192.1283	3.57		
3	4.413	154.2668	3.43		
	4.681	112.2400	2.49		
	4.683	94.1860	1.75		
	4.726	11.3554	0.20		
	6.255	49.7648	1.11		
	6.255	69.8084	1.30		
	8.868	63.5794	1.18		
	9.839	270.9656	4.83		

Single Injection Report

Agilent Technologies

Name	RT	Area	Peak Area Percent	Unit	Concentration []
	9.839	29.1615	0.54		
	11.652	34.1563	0.64		
	11.653	40.0921	0.89		
	13.285	297.7824	6.62		
	13.292	1931.3670	34.39		
4	13.292	1306.8191	24.30		
	14.668	51.1434	0.95		
	15.903	108.3426	1.93		
	15.905	39.4806	0.88		
	20.353	69.5240	1.24		
	22.053	32.7977	0.58		
	23.483	34.6186	0.64		
7	23.938	38.5954	0.69		
	23.939	17.3902	0.32		
8	24.764	12.8520	0.29		
	24.975	30.5982	0.57		
	25.003	11.4010	0.20		
	25.532	11.6599	0.21		
	26.250	16.6855	0.31		
	26.251	12.4519	0.22		
	27.612	44.9525	0.84		
9	27.613	84.1694	1.50		
	29.656	15.3395	0.27		
	29.657	14.2017	0.26		
10	29.660	29.6545	0.66		
	30.656	67.0255	1.49		
	30.656	53.4195	0.99		
	30.657	71.7533	1.28		
	30.946	18.2383	0.32		
	31.802	11.0830	0.25		
	31.804	46.2881	0.82		
	31.805	34.9636	0.65		
	34.021	16.5816	0.37		
	34.333	16.7154	0.30		
	34.807	7.9268	0.14		
12	36.661	71.3704	1.59		
	36.661	126.1906	2.25		

Single Injection Report

Agilent Technologies

Name	RT	Area	Peak Area Percent	Unit	Concentration []
	36.662	190.6141	3.55		
	38.917	13.9219	0.26		
	38.918	9.1784	0.16		
	40.435	15.6339	0.35		
	42.124	39.1520	0.70		
	42.124	42.0307	0.78		
	42.124	18.6802	0.42		
	44.238	11.0982	1.30		
	44.238	8.9815	0.17		
	44.239	36.4176	0.65		
	46.197	16.3244	0.36		
	46.832	22.1752	0.41		
	46.842	58.7509	1.31		
	47.668	57.2666	1.27		
	50.076	20.8737	0.46		
	50.081	19.9923	0.37		
	52.014	67.1903	1.49		
	52.650	15.7788	0.35		
	53.907	12.5616	0.22		
	53.907	11.8212	0.22		
13	54.238	95.3523	2.12		
	54.244	98.0522	1.75		
	54.245	130.3869	2.42		
14	54.851	686.2865	15.25		
	54.852	76.4597	1.36		
	54.853	66.7260	1.24		
	55.909	7.3229	0.14		
	56.248	14.8228	0.26		
	56.248	15.2159	0.28		
	56.733	29.5830	0.66		
	56.734	63.8937	1.14		
	56.734	87.0809	1.62		
	58.193	19.5858	0.44		
	58.438	6.0428	0.11		
	59.876	10.9262	0.24		
	60.942	41.4178	0.92		

Single Injection Report

Agilent Technologies

MWD1A,Sig=250,4 Ref=off

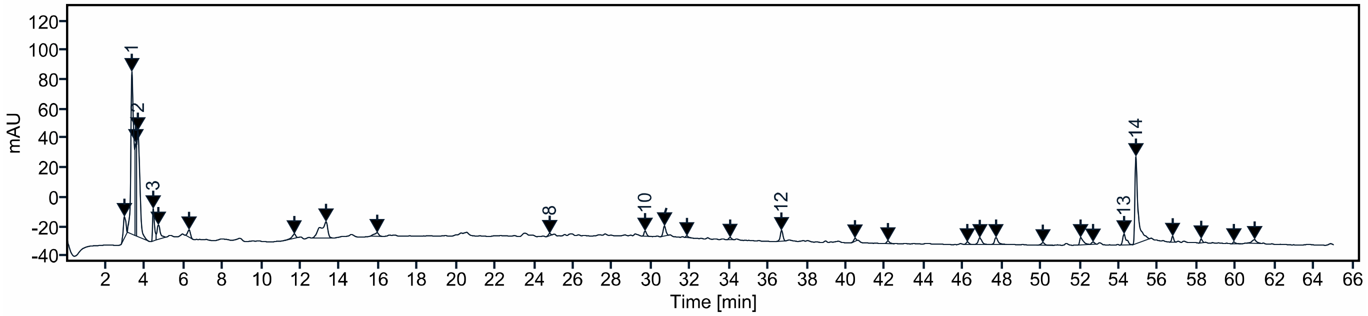

MWD1B,Sig=270,4 Ref=off

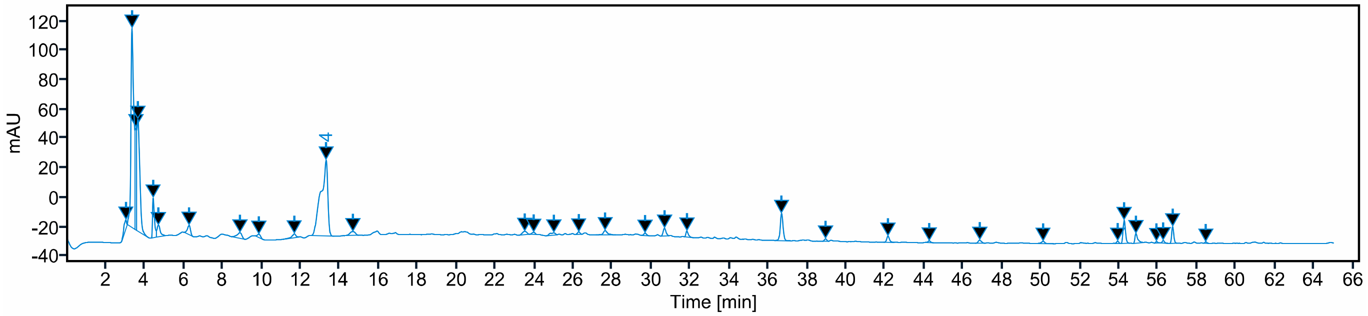

MWD1C,Sig=290,4 Ref=off

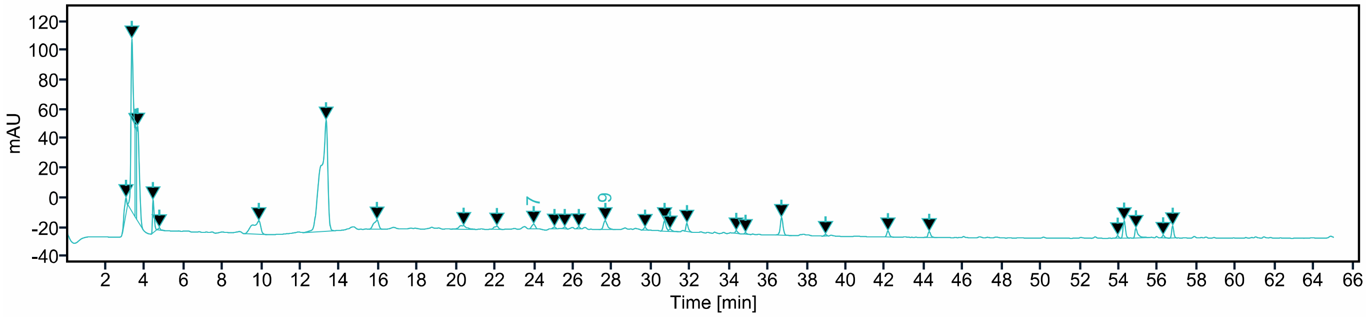

MWD1D,Sig=350,4 Ref=off

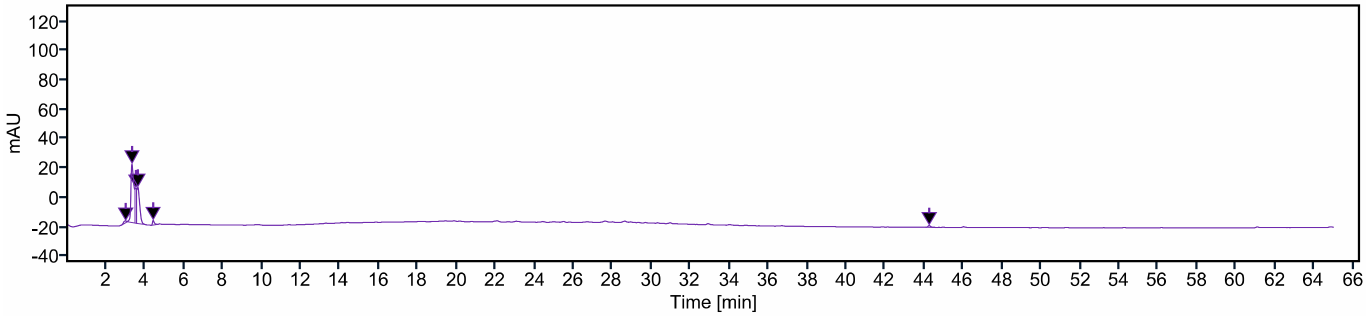

MWD1E,Sig=380,4 Ref=off

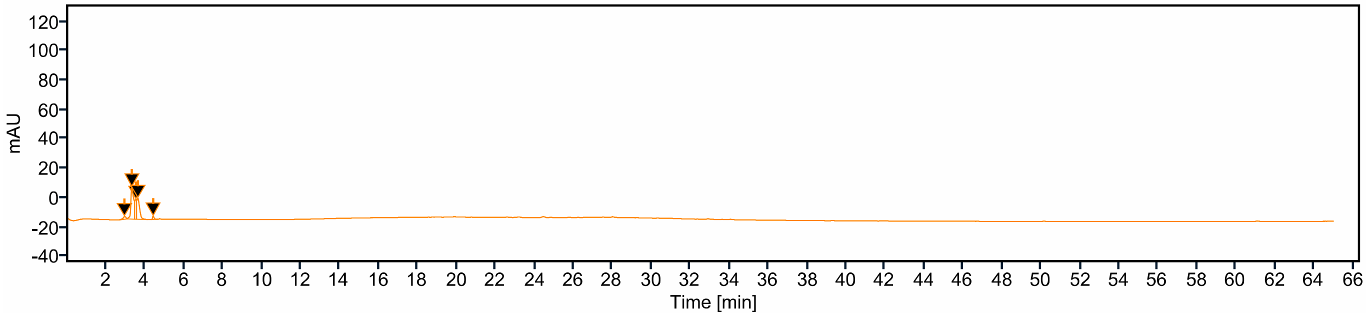

Supplement: Supplementary file 1 [file molecules-29-00117-s001.zip › molecules-2641918-supplementary.pdf]
